# Influence on number of top-ups after implementing patient controlled epidural analgesia: A cohort study

**DOI:** 10.1371/journal.pone.0186225

**Published:** 2017-10-18

**Authors:** Ganapathy van Samkar, Henning Hermanns, Philipp Lirk, Markus W. Hollmann, Markus F. Stevens

**Affiliations:** Department of Anaesthesiology, Academic Medical Centre, Amsterdam, the Netherlands; University Medical Center Goettingen, GERMANY

## Abstract

Postoperative epidural analgesia often needs rate readjustment using top-ups. Patient-controlled epidural analgesia (PCEA) is said to reduce the requirement of epidural top-ups when compared to continuous epidural analgesia (CEA). We compared CEA and PCEA in major thoracic and abdominal surgery, in a cohort study. The primary endpoint was the required number of epidural top-ups. Secondary endpoints were pain scores, side effects and workload differences. We analysed 199 patients with CEA and 187 with PCEA. Both groups had similar pain scores. The total number of top-ups was 75 in 57 patients (CEA) versus 20 top-ups in 18 patients (PCEA). (*p* = 0.0001) Sedation tended to occur more frequently in patients with CEA versus PCEA, 5.5% vs 1.6% (*p* = 0.05). Implementation of PCEA led to a decreased number of top-ups, fewer side-effects and decreased use of the postoperative care unit.

## Introduction

Epidural analgesia is regularly applied perioperatively for major abdominal or thoracic surgery.[1, 2] However, epidural analgesia can have a failure rate as high as 30%, frequently requiring re-adjustment by increasing the speed of infusion and/or top-ups with a bolus of local anaesthetic.[3–5] Nevertheless, top-ups with larger doses of local anaesthetics and/or opioids can cause hemodynamic or respiratory depression and therefore require intensified monitoring.

A refinement of continuous epidural analgesia (CEA) is patient controlled epidural analgesia (PCEA) where a basal epidural infusion rate can be supplemented by an on demand bolus. The efficacy of PCEA has already been investigated in numerous clinical studies, and confirmed in a systematic review.[6–14] PCEA induced superior analgesia with fewer side effects and a decrease in drug requirement. However, many hospitals and anaesthesiologists continue using CEA for reasons of simplicity, scarcity of PCEA pumps and intricacy of handling of these pumps. In our institution CEA was the standard of care before we introduced PCEA. Monitoring of an epidural top-up can be challenging to manage, given the logistics of a large hospital and teaching centre: response time, transport time, time to contact a physician to do the top-up, and assessment, supervision and monitoring time. We investigated whether the introduction of PCEA infusion pumps on the regular postoperative wards decreased the need for postoperative top-ups. Thus—in contrast to previous studies—the primary aim of this study was to reduce the number of top-ups after implementation of PCEA. Secondary outcome measures were: pain score (numeric rating scale, NRS), side effects (sedation, itching, motor block, nausea and vomiting) and calculated hours of differences in workload. Our hypothesis was that PCEA would reduce the number of top-ups, side effects and workload. Further, it could lead to a reduced duration of use of epidural analgesia.

## Materials and methods

In this retrospective cohort study, we investigated adult patients who had undergone thoracic and upper abdominal surgery during 2012–2013. The institutional medical ethics committee provided a waiver (W14-051 # 14.17.005) for this anonymized investigation. Patient consent was not required, as data used for this cohort analysis was already present, and patients were not subjected to study measures. Epidural catheters were placed preceding the induction of general anaesthesia by a consultant or a registrar in anaesthesia with adequate experience, and proper placement was confirmed, according to local standard operating procedures. The epidural catheter was used for analgesia during the operation, infusion of bupivacaine 0.25% at the rate of 8–10 ml per hour. Patients received only standard CEA for nine months in 2012. (N = 199). After educating all care givers about the technique of PCEA, patients received PCEA for nine months in 2013 (N = 187). Itching (tolerable or needing medication), motor weakness (Bromage score), sedation (Ramsay score) and nausea (tolerable or needing medication) were scored. For safety reasons (e.g. monitoring of hypotension and respiratory depression), top-ups (top-ups: lidocaine 1%, dosed at 1mg/kg body weight) were given by a physician under basic monitoring (by means of non-invasive blood pressure, ECG and saturation). In patients not needing top-ups, the standard rate of epidural infusion was as per protocol (see below). Primary and secondary epidural failures were scored in both groups: primary failure was defined as: the epidural was not working immediately after the operation in spite of top-up, and secondary failure was defined as: initially good analgesia, but in the course of time a failed epidural (no analgesia) in spite of top-ups. Peak NRS scores were registered before top-ups in both groups. Both, primary and secondary failures were included in an intention-to-treat analysis. Workload was calculated as the amount of time spent by medical professionals to treat inadequate postoperative epidural analgesia.

### CEA protocol

Standard epidural medication was bupivacaine 0.125% with 1 microgram sufentanil per ml solution. In patients older than 70 years or weighing less than 60 kg, sufentanil was omitted from the epidural. The epidural pump was set at a constant speed of 10 millilitres per hour (ml/hr). The rate was increased by 2 ml/hr if indicated by pain scores (see below), after a top-up dose. Maximum dose was 0.3 mg/kg per hour of bupivacaine. The rate was decreased by 2 ml/hr if analgesia was adequate or in the presence of hypotension.

### PCEA protocol

Epidural solution was identical to the CEA protocol. The epidural infusion speed was 6 ml/hr with a patient controlled bolus of 2 ml and a lockout time of 20 minutes. The maximum bupivacaine dose was defined as 0.3 mg/kg per hour. In case of inadequate analgesia the bolus was primarily increased from 2 ml to 4 ml. In case of arterial hypotension the rate was decreased by 2 ml/hr (as above).

### Both groups

Standard additional medication (unless they were contraindicated) included acetaminophen 4 grams (g) daily in 4 doses, and diclofenac 150 milligrams (mg) daily in 3 doses or dipyrone 4 grams (g) daily in 4 doses. We used the following pump: BBraun PerfusorSpace with special module for PCEA. This enabled us to use the same pump for continuous epidural and patient controlled epidural analgesia by adding an extra module with button for patient control.

### Pain scores

The first pain scores were routinely taken at the post anesthesia care unit (PACU). After transferring the patients to the surgical wards, the level of epidural analgesia was judged by the staff of the acute pain service daily, and if inadequate (resting NRS score above 4 in the operated location, inadequate block height) the patient received an epidural top-up bolus with lidocaine 1%, dosed at 1 mg/kg. This was done after transferring the patient to the PACU under extended hemodynamic and neurologic monitoring, because of the complexity of the patient population with underlying diseases in a university hospital.This is partially reflected in ASA class distribution in Table 1, bearing in mind that a pancreatic resection or transthoracic esophageal resection remains a high risk procedure even in patients categorized as ASA 1 and 2. In addition, NRS scores were documented by the ward personnel 3–4 times daily, and if scores were above 4, the acute pain service was called. Patients could also alert the nurses if they felt uncomfortable due to pain. In both groups, successful top-ups were followed by an increase in basic epidural infusion speed (in case of pain during rest). In the PCEA group pain during activity was treated by an increase in bolus dose. Total failure of epidural analgesia (insufficient effect of top-up) was followed by removal of the epidural catheter and the initiation of patient controlled analgesia with morphine (PCA).

**Table 1:** Patient and treatment characteristics.

	CEA* N = 199	PCEA† N = 187	P-Value
Male/Female N (%)	63/136 (32/68)	45/142 (24/76)	0.131‡
Mean Weight in kg SD	73 15	73 14	0.824§
Median age IQR	60 (47–68)	62 (52–70)	0.566
ASA class** N (%)			0.08‡
1	55 (28)	70 (38)	
2	122 (61)	94 (50)	
3	22 (11)	23 (12)	
**Operation type N (%)**			¶
PPPD, pancreatic surgery	18 (9)	42 (23)	0.000
Thoracic oesophagus resection	25 (13)	7 (4)	0.000
Trans hiatal oesophagus resection	8 (4)	1 (0.5)	0.04
Laparotomy	58 (29)	45 (24)	0.30
Debulking tumour load	31 (16)	42 (23)	0.16
Wertheim	25 (13)	18 (11)	0.42
Hemihepatectomy	10 (5)	12 (6)	0.66
Liver hilus resection	3 (2)	0	0.25
Gastrectomy	3 (2)	6 (3)	0.32
Pelvic exenteration	3 (2)	0	0.25
Liver segment resection	4 (2)	5 (3)	0.74
Colonic surgery	11 (6)	8 (4)	0.64
**Level of Epidural N(%)**			
T6-T7	24 (12)	11 (6)	0.05
T7-T8	36 (18)	20 (11)	0.04
T8-T9	41 (21)	31 (17)	0.36
T9-T10	38 (19)	35 (19)	1
T10-T11	18 (9)	46 (25)	0.000
T11-T12	5 (3)	17 (9)	0.007
T12-L1	5 (3)	6 (3)	0.76
L1-L2	28 (14)	20 (11)	0.36

**ASA class, American Society of Anesthesiologists physical status classification

*CEA, Continuous Epidural Analgesia

†PCEA, Patient Controlled Epidural Analgesia.

‡2 sided Pearson Chi Square test.

§t Test Bias Corrected Accelerated.

¶ Fisher exact two tailed.

### Endpoints

Primary endpoint was the cumulative frequency of top-up rescue interventions per therapy group throughout the entire period of postoperative epidural analgesia. Secondary endpoints were: NRS pain scores, side effects (hypotension, nausea, vomiting, itching, motor weakness, sedation) and estimated differences in workload. Hypotension was generally defined as: when mean arterial pressure decreased more than 20% from the normal mean arterial pressure of the patient as commonly measured in normal circumstances. Additionally, for the workload calculation, we measured the average time involved in a top-up of a surgical ward patient. Including transport, this was 2.5 hours per patient. (30 min transport to and from the PACU, 2 hours observation including top-up of epidural on PACU).

### Statistics

SPSS version 22 (IBM software, New York, USA) was used to analyze our data. Normality of distribution was evaluated using the Shapiro-Wilk test. Student’s t-test or Mann-Whitney U test was used to calculate differences in mean or median where appropriate. Continuous data not normally distributed were analyzed by a Kruskal-Wallis test and if significant followed by Mann-Whitney U test. Categorical data and frequencies were analyzed by Fisher’s exact test. Confidence intervals of 95% are given where appropriate, otherwise data are presented as means with standard deviations (SD) or median with interquartile range (IQR), respectively. A *p-*value of < 0.05 was considered statistically significant.

## Results

**Fig 1:**
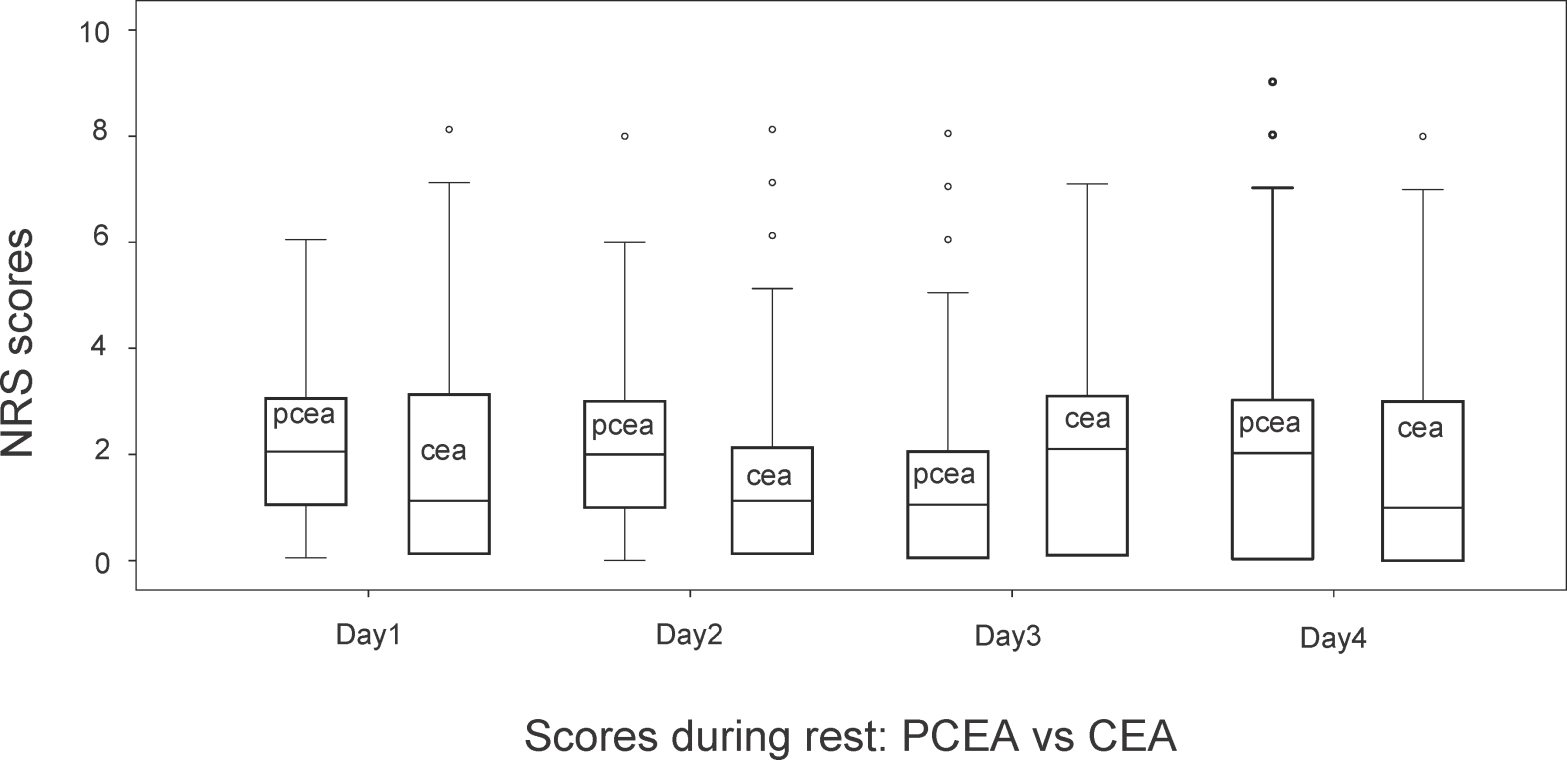
NRS* resting pain scores postoperative day 1 to day 4, CEA† (N = 199) vs PCEA‡ (N = 187). *NRS, Numeric rating scale of pain. †CEA, continuous epidural analgesia, ‡PCEA, patient controlled epidural analgesia. Depicted in the boxes are resting postoperative pain scores of 4 days in patients with continuous and patient controlled epidural analgesia. Top of box is third quartile, bottom is first quartile. The horizontal line in box is median value; whiskers at the end of lines are minimum and maximum values. Dots are outliers.

**Fig 2:**
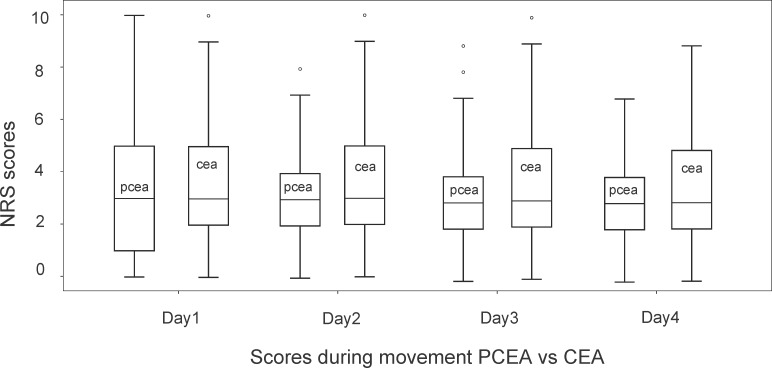
NRS* pain scores during movement postoperative day 1 to day 4, CEA† (N = 199) vs PCEA‡ (N = 187). *NRS, Numeric rating scale of pain. †CEA, continuous epidural analgesia, ‡PCEA, patient controlled epidural analgesia. Depicted in the boxes are resting postoperative pain scores of 4 days in patients with continuous and patient controlled epidural analgesia. Top of box is third quartile, bottom is first quartile. The horizontal line in box is median value; whiskers at the end of lines are minimum and maximum values. Dots are outliers.

A total of 386 patients were analysed from 2012 to 2013: 199 in the CEA and 187 in the PCEA group. There were no significant differences between the two groups regarding age, weight, distribution of sex. Regarding type of surgery, there were significantly more oesophageal resections with CEA. On the other hand, significantly more patients underwent pylorus preserving pancreato duodenectomy with PCEA. More than 80% of epidurals were placed at thoracic level in both groups. (Table 1) In the group of patients with CEA, 75 top-ups were necessary, compared to 20 in the PCEA group (p = 0.0001). There were no significant intergroup differences in NRS scores on Postoperative day 1 to 4. (Figs 1 and 2). Peak NRS scores before top-up did not differ between groups.

**Fig 3:**
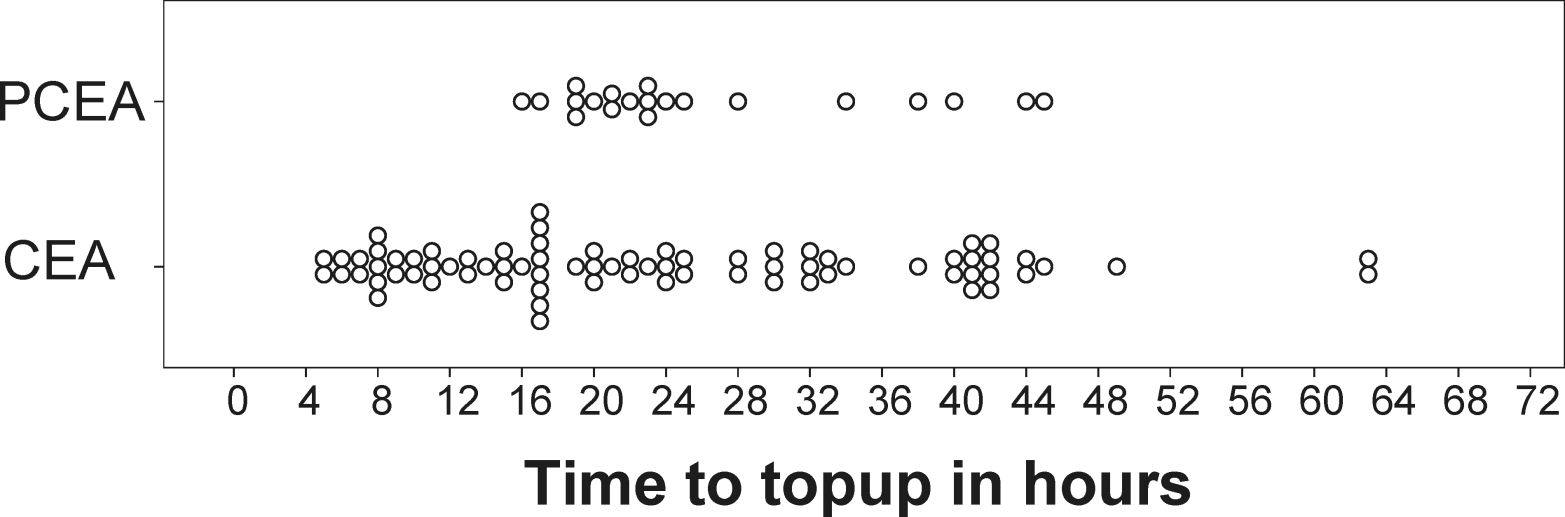
Top-up administration in CEA and PCEA groups. †CEA, continuous epidural analgesia, ‡PCEA, patient controlled epidural analgesia. Each circle represents one top-up. The time-interval is given in hours. In order to visualize each patient data points were mildly shifted in time and stacked to improve readability of the figure.

Primary and secondary endpoints are compared in Table 2. Itching, nausea and motor weakness was not significantly different between groups. The timings of top-ups are represented in Fig 3.

**Table 2:** Comparison of endpoints between CEA* and PCEA†.

	CEA* (N = 199)	PCEA† (1879(18((N = 187)	*p* value ‡
Total number of patients with Top-ups N (%)	57 (28.6)	18 (9.6)	0.0001
Requiring One top-up N	41	16	0.001
Two top-ups N	14	2	0.004
Three top-ups N	2	0	0.49
Mean days duration epidural analgesia (SD)	3.3 (1.5)	3 (1)	0.07
Median Peak NRS scores (95% CI)	8 (7–8)	8 (7–8)	0.75
Primary failure of epidural	6	1	0.12
Secondary failure of epidural	2	4	0.44
**Side effects**			
Itching untreated N (%)	4	3	0.57
treated	4	2	
Nausea untreated N (%)	11	10	0.52
treated	14	9	
Motor weakness total N (%)	28	19	0.27
Bromage level 2	13	9	
Bromage level 3	11	9	
Bromage level 4	4	1	
Sedation total N (%)	11 (5.5)	3 (1.6)	0.05
Ramsay score 3	3	2	
Ramsay score 4	2	1	
Ramsay score 5	6	0	
Any side effect (%)	72 (36.1)	46 (24.5)	0.02

*CEA, continuous epidural analgesia.

†PCEA, Patient controlled epidural analgesia.

‡ Fisher exact.

Post-hoc exclusion of pancreatoduodenectomies and oesophagectomies to control for non-random distribution of these procedures between groups resulted in 148 patients with CEA and 137 patients with PCEA with 32 top-ups in the CEA group, and 16 top-ups in the PCEA group (p = 0.03). Thus, the difference remains significant even in the patients with presumably less painful operations.

Table 3 presents reasons for decreasing the rate of the epidural infusion. We decreased rates in 17 patients (8.5%) in the CEA group and in 3 patients (1.6%) in the PCEA group (p = 0.002).

**Table 3:** Rate adjustment due to side effects.

Number of patients for whom:	CEA* (N = 199)	PCEA† (N = 187)	*p* value‡
rate was decreased due to motor blockade	5	3	0.5
rate was decreased due to sedation	5	0	0.02
rate was decreased due to arterial hypotension	7	0	0.006
Total N (%)	17 (8.5)	3 (1.6)	0.002

*CEA, continuous epidural analgesia.

†PCEA, Patient controlled epidural analgesia.

‡Fisher exact

### Workload calculation

In our hospital, the average time spent on the monitoring ward, was 2 hours. Transport to and from the surgical and gynecological wards required on average 30 minutes per patient. This sums up to an average workload per (patient) top-up of 2.5 hours in our setting. We had 20 top-ups in our PCEA group of 187 epidurals, and 75 in our CEA group of 199 patients., 20 top-ups result in 50 hours per 187 patients receiving PCEA, this is 16 minutes per patient in this group. 75 top-ups result in 187.5 hours per 199 patients receiving CEA, this is 56.5 minutes per patient in this group.

## Discussion

Our main finding in this retrospective cohort study was that the use of PCEA significantly reduced the number of patients requiring top-ups, while NRS scores did not differ between groups. The total numbers of top-ups in our study are in accordance with other studies: a Swedish study encompassing seven years of PCEA and 4912 epidurals had a failure rate of 11%, resulting in termination of the epidural.[15] Recent literature gives a failure rate of up to 30% in CEA epidurals. [3, 16]

Our study investigates the effect of implementation of PCEA on the total number of rescue top-ups, which form a logistically important and costly aspect of postoperative epidural analgesia. The finding that the PCEA group did *not* improve pain scores is in contrast to other studies.[7, 9] However, most studies comparing PCEA and CEA were done before the introduction of multimodal pain concepts. Thus, the fact that all patients continued preoperative pain medication with the addition of acetaminophen and diclofenac or dipyrone may have also worked in favour of the pain scores in the CEA group. Well in accordance to the quoted comparative studies between CEA and PCEA, we noticed more side effects in the CEA group. There was a significant difference between groups, in the number of patients requiring reduction of infusion rate due to side effects such as sedation, motor block or hypotension. The degree of sedation was considerably lower in patients with PCEA. Also, fewer patients were sedated. Since our pumps only register drug consumption over the last 4 hours, unfortunately we were not able to obtain results regarding the applied doses, but it is likely that the increased percentages of side effects in the CEA group were caused by high local anaesthetic and opioid doses applied. Regarding hypotension and possible respiratory complications after epidural analgesia, these frequencies may be under reported. Due to the retrospective nature of the study, we can only show the actual documentation of these events. Furthermore, the incidence of hypotensive episodes may have been influenced by the the epidural level. In the CEA group midthoracic epidural levels (T6-T8) were more frequent than in the PCEA group where low thoracic levels(T10-T12) were more frequent. Therefore these are limitations of the study.

Our PCEA algorithm is rather conservative, and there are studies with more successful algorithms; especially those with integrated mandatory and automatic bolus.[17] Nevertheless, we noticed a significant improvement in our in-hospital logistics after the introduction of PCEA pumps. Perhaps the feeling of being in control positively adds to the success of PCEA, as suggested in an earlier publication.[18]

More than a decade ago, Schuster and co-workers calculated the cost of PCEA and demonstrated that most of the money is spent on staff costs, although in their calculation they did not include expenses for top-ups at medium- or high-care units.[19] In an earlier study of 6349 patients, Brodner and co-workers demonstrated significant cost savings due to the implementation of a multimodal pain management including PCEA.[20] Furthermore, in the last decennium, the percentage of staff cost in developed countries increased further while drug and material costs tended to decrease. In our hospital the transport and admittance of patients for epidural top-ups is not only time-consuming, but because of its urgent character it cannot be scheduled or planned and can create logistic problems for the ward, transport service and the postoperative care unit. The introduction of PCEA did significantly ameliorate this problem.

In our hospital, we calculate 16 minutes per patient in the PCEA group versus 56.5 minutes in the CEA group. Even though this is specific to our hospital and may not reflect the situation in other hospitals, top-ups are always time consuming, and efficiency is welcome. Top-ups are often done in the wards, but even then if the frequency of top-ups can be drastically reduced, it is beneficial to workload.

Thus, not only patient comfort and success rate were increased (decrease in sedation and less top-ups) but also hospital investment of costly urgent medium or high care space.

Our study has several limitations: Patients with oesophageal and pancreatic surgery were not equally distributed between cohorts. However, excluding these patients in a post-hoc analysis revealed even in the remaining and presumably less painful operations, a significant difference in the number of top-ups between groups (p = 0.03). In this subgroup the number of side-effects leading to changes in management was significantly more in the in CEA group than in the PCEA group. Thus, the non-randomized nature of the study leads to an uneven distribution of operations between groups, but the results were robust enough, when controlled for the uneven distribution. Furthermore, due to the nature of the study (not an RCT, no blinding) there are many possible causes of bias: the effect of the PCEA may be due to the psychological factor of “self-control”, resulting in less complaints, nurses may call the pain service earlier in case of CEA, or delay because of the hassle involved in a top-up dose. Irrespective of whether the effect of PCEA was caused by psychological or pharmacologic factors, in clinical practice it will have a benefit. Whatever bias may have been involved, it did not seem to result in a significant difference in NRS scores between groups. Although our results may need validation in a prospective randomized trial, we demonstrated for the first time that PCEA could reduce the frequency of top-ups and thereby reduce inconvenience for the patient, workload for the staff and costs for the hospital.

## Conclusion

We conclude that PCEA can reduce the frequency of top-ups and side effects, compared to CEA. This may lead to reduced logistic workload and hospital costs.

## Supporting information

S1 File Database pcea vs cea 23 aug for plosone. (XLSX)

S1 Table Ramsay score. (DOCX)

S2 Table Bromage score. (DOCX)
