# Supplementary material for: Influence on number of top-ups after implementing patient controlled epidural analgesia: A cohort study
Source: PLoS One. 2017 Oct 18;12(10):e0186225. doi: 10.1371/journal.pone.0186225 (PMC5646815; doi:10.1371/journal.pone.0186225)
Supplement: S1 Table — (DOCX) [file pone.0186225.s002.docx]

|  | Ramsay |  |
| --- | --- | --- |
| Awake | 1 | Anxious, agitated, restless |
|  | 2 | Cooperative, oriented, tranquil |
|  | 3 | Responsive to commands only |
| Asleep | 4 | Brisk response to light glabellar tap or loud auditory stimulus |
|  | 5 | Sluggish response to light glabellar tap or loud auditory stimulus |
|  | 6 | No response to light glabellar tap or loud auditory stimulus |
