# Supplementary material for: Influence on number of top-ups after implementing patient controlled epidural analgesia: A cohort study
Source: PLoS One. 2017 Oct 18;12(10):e0186225. doi: 10.1371/journal.pone.0186225 (PMC5646815; doi:10.1371/journal.pone.0186225)
Supplement: S2 Table — (DOCX) [file pone.0186225.s003.docx]

| Bromage | Criteria | Degree of block |
| --- | --- | --- |
| 1 | Free movement of legs and feet | Nil |
| 2 | Just able to flex knees with free movement of feet | Partial |
| 3 | Unable to flex knees, but with free movement of feet | Almost complete |
| 4 | Unable to move legs or feet | Complete |
